# We Need a Better Medical Law

**Published:** 1894-11

**Authors:** T. D. Wooten

**Affiliations:** President Board of Regents, University of Texas; Austin, Texas


### We Need a Better Medical Law.

---

Austin, Texas, November 1, 1894.

*Editor Texas Medical Journal.*

In all civilized countries, the medical profession have been and are the conservators of public health, the world over. There are some matters of public interest to Texas and to all Texans, and of special interest and importance to her medical men as concerning their character, reputation and dignity, and their obligations to the public, to which I wish respectfully to call attention through the pages of the Journal. I would solicit their influence in abating the present very unfavorable attitude in which the profession is placed in relation to the public and its welfare, in consequence of our very defective and unsatisfactory medical law.

The State of Texas, by her wise legislators, has honored the medical profession and done herself credit is establishing a Medical Department of our State University, and so far as means would allow it has been made a medical school of the first class. The curriculum is of a high order, and the course of study is a graded three years course, of seven months each session, and will compare favorably with any medical school in this country. The equipment, laboratories and buildings are a credit and an honor to the State and to our profession; and, let me say, it deserves at the hands of the profession of Texas a fostering care and interest; and let me say here, that the medical profession of Texas will be recreant to its honor and dignity if it does not come to the front in aid of the State in its proper protection and maintenance.

To do our duty in these matters, the profession of Texas should insist, since the State has established the medical school and has made it almost free to the professional students of Texas, at any rate, cheaper than any school of the kind in the United State, that the legislature should abolish in toto the present law regulating the practice of medicine, and require, in the future, that no one will be permitted to practice medicine except those who have graduated at a school of like requirements for graduation, say of three years graded course of medical instruction of not less than six months sessions, ours being seven. We should not compel our students to go to our school, but since the State has made the course almost free to her students, the State can, with very just propriety, require a like course as she has provided for them.

The present examining boards should and must be abolished. At the last session of the medical school, there were two students who were unable, on examination, to pass from the first year to the second. They quit the school and went before a board, and got licenses to practice, and are now practicing or are trying to practice. The profession of Texas should not submit willingly to this state of things. Whilst I would not recommend legislation that would disturb the status of those who have qualified to practice medicine under existing laws, it is due from the medical profession to insist that a law shall be passed requiring that all doctors who practice medicine in the future in Texas shall have received a medical education equal in degree to the one provided by the State. Respectfully,

T. D. WOOTEN, M. D.,
President Board of Regents, University of Texas.

